# Transcription Factor VvDREB2A from *Vitis vinifera* Improves Cold Tolerance

**DOI:** 10.3390/ijms24119381

**Published:** 2023-05-27

**Authors:** Lixia Hou, Qiqi Wu, Xiaomin Zhu, Xiangyu Li, Xinxin Fan, Mengling Hui, Qing Ye, Guangchao Liu, Xin Liu

**Affiliations:** Key Lab of Plant Biotechnology in University of Shandong Province, College of Life Science, Qingdao Agricultural University, Qingdao 266109, China; houlixia@qau.edu.cn (L.H.); wuqiqi@stu.qau.edu.cn (Q.W.); 20202206043@stu.qau.edu.cn (X.Z.); 20212206023@stu.qau.edu.cn (X.L.); fxx15020186726@163.com (X.F.); 20210202470@stu.qau.edu.cn (M.H.); 201801052@qau.edu.cn (Q.Y.); gcliu@qau.edu.cn (G.L.)

**Keywords:** VvDREB2A, *Vitis vinifera* L., cold stress, ROS, raffinose family oligosaccharides, cold stress-related genes

## Abstract

Low temperatures restrict the growth of the grapevine industry. The DREB transcription factors are involved in the abiotic stress response. Here, we isolated the *VvDREB2A* gene from *Vitis vinifera* cultivar ‘Zuoyouhong’ tissue culture seedlings. The full-length *VvDREB2A* cDNA was 1068 bp, encoding 355 amino acids, which contained an AP2 conserved domain belonging to the AP2 family. Using transient expression in leaves of tobacco, VvDREB2A was localized to the nucleus, and it potentiated transcriptional activity in yeasts. Expression analysis revealed that *VvDREB2A* was expressed in various grapevine tissues, with the highest expression in leaves. *VvDREB2A* was induced by cold and the stress-signaling molecules H_2_S, nitric oxide, and abscisic acid. Furthermore, *VvDREB2A*-overexpressing *Arabidopsis* was generated to analyze its function. Under cold stress, the *Arabidopsis* overexpressing lines exhibited better growth and higher survival rates than the wild type. The content of oxygen free radicals, hydrogen peroxide, and malondialdehyde decreased, and antioxidant enzyme activities were enhanced. The content of raffinose family oligosaccharides (RFO) also increased in the *VvDREB2A*-overexpressing lines. Moreover, the expression of cold stress-related genes (*COR15A*, *COR27*, *COR6.6*, and *RD29A*) was also enhanced. Taken together, as a transcription factor, VvDREB2A improves plants resistance to cold stress by scavenging reactive oxygen species, increasing the RFO amount, and inducing cold stress-related gene expression levels.

## 1. Introduction

Grapevine (*Vitis vinifera* L.) is a commercially grown fruit tree planted worldwide. Most of the commonly planted grapevine varieties in China are Eurasian, which are sensitive to low-temperature stress and have poor cold tolerance [1]. Moreover, frost during the winter and early spring seriously affects the yield and quality of grapevines in northern China. Extremely cold weather has occurred frequently in recent years, and frost damage, as a “bottleneck”, has greatly restricted the development of the grapevine industry.

Some progress has been made in understanding the action mechanism of the plant’s response to cold stress. The low-temperature signaling pathway in plant cells is transmitted in a particular sequence: membrane receptors—Ca^2+^—protein kinases—transcription factors—cold-related genes—physiological and biochemical reactions—cold resistance [2]. Under cold stress conditions, plants regulate gene expression through a complex signal transduction network, ultimately increasing the content of osmotic substances, regulating osmotic pressure, scavenging reactive oxygen species (ROS), and stabilizing cell membranes to alleviate cell damage at low temperatures [3,4]. Transcription factors are the pivots in the response to low temperatures. Among these transcription factors, CBF/DREB binds to the DRE/CRT motif, which activates the transcriptional expression of the downstream COR gene and participates in the resistance to cold stress [5,6].

The DREB transcription factor plays a crucial roles in the abiotic stress response. It consists of a highly conserved ERF or AP2 domain, about 60 amino acids, which is in charge of the specific binding with DNA [7]. According to the conserved domain, the DREB transcription factor family members can be divided into six subfamilies (A1–A6), and many studies have been conducted on the A1 and A2 subfamilies [8,9]. DREB/CBF research in the A1 subfamily has concentrated on low temperatures [10,11,12]. Many *DREB1/CBF* genes have been identified in *Arabidopsis*, *Malus domestica*, *Medicago truncatula*, *Hordeum vulgare*, *Triticum aestivum*, and *Zea mays*, and they play a critical role under cold stress. Research on the A2 subfamily of DREB2 transcription factors has mainly focused on the resistance to salt, drought, and heat stress [10,13]. However, this classification is not absolute and the classification in different literatures is not completely consistent. Some DREB2 are not only involved in drought stress, but also in cold tolerance [14,15,16].

Thirty-eight VvDREB members have been identified from the entire grapevine genome. These members were divided into six groups (A1–A6), as in the *Arabidopsis* DREBs [17]. Many studies have focused on the A1 subfamily, revealing that overexpression of *VvCBF4/VvDREB1D* in wine grapes improves cold resistance [18]. The grapevine *VrCBF1/DREB1B* and *VrCBF4/DREB1D* overexpression in *Arabidopsis* improves cold tolerance through increasing the activities of protective enzymes and activating the expression of the cold response genes *COR15A*, *COR6.6*, *COR47*, and *RD29A* [19]. Nevertheless, the function of the DREB A2 subfamily members in the grapevine has rarely been reported, and the mechanism has not been revealed.

In this study, we reported a DREB A2 subfamily member, *VvDREB2A*, from the cold-tolerant grapevine ‘Zuoyouhong.’ Its characteristics were identified, and the expression pattern was detected. Furthermore, we also generated *VvDREB2A* ectopic overexpressing *Arabidopsis* lines, observed the phenotypes after cold treatment, and measured some cold-related physiological indicators and the expression level of cold-response genes. The data reveal the function and mechanism of VvDREB2A under cold stress and contributes to providing candidate genes for grapevine breeding.

## 2. Results

### 2.1. Isolation and Sequence Analysis of VvDREB2A

**Figure 1:**
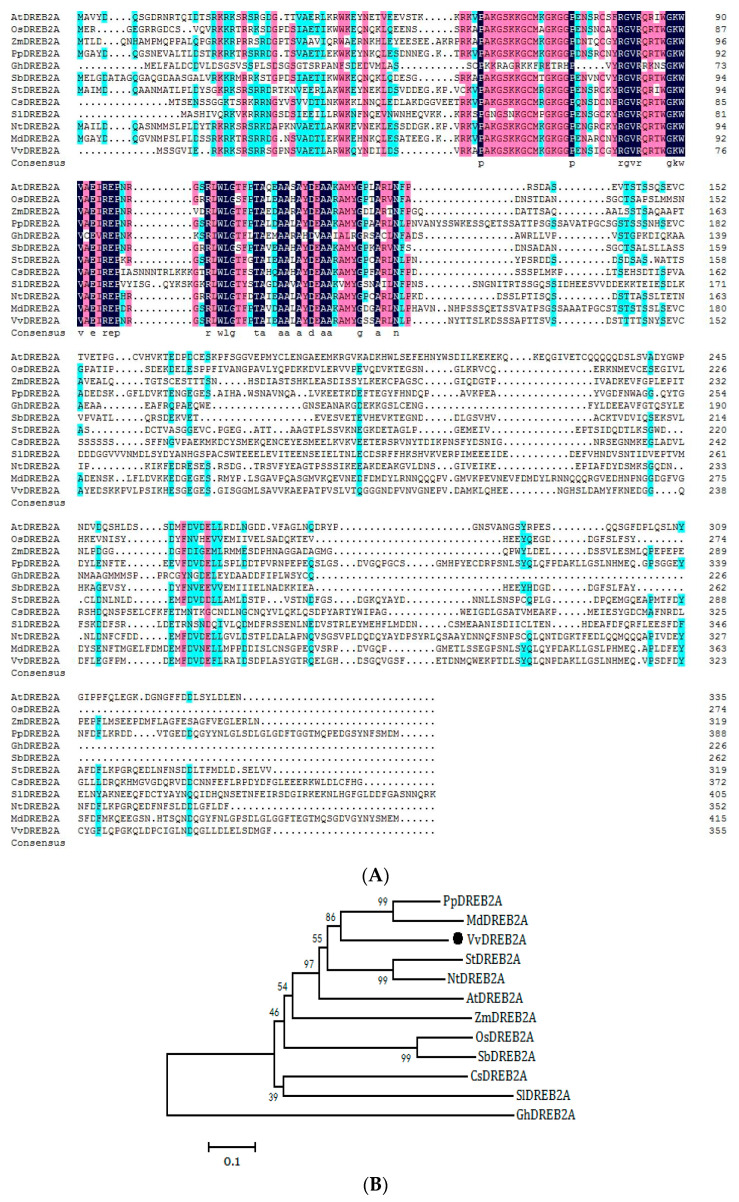
VvDREB2A sequence analysis. (**A**) Alignment of amino acid sequences of VvDREB2A and DREB2A from other species (GenBank Accession numbers: AtDREB2A: NP_196160; OsDREB2A: XP_025878770; ZmDREB2A: NP_001105876; PpDREB2A: XP_020413813; GhDREB2A: NP_001314301; SbDREB2A: XP_002457289; StDREB2A: NP_001305480; CsDREB2A: XP_004148625; SlDREB2A: XP_010327719; NtDREB2A: XP_009788693; MdDREB2A: NP_001280947; VvDREB2A: AZI71501). (**B**) Phylogenetic tree of VvDREB2A and DREB2A from other species.

First, we used the AtDREB (AT2G40340) amino acid sequence from TAIR to obtain the homolog in grapevine using WU-BLAST. The homologous sequence from the grapevine cultivar ‘Pinot Noir’ was XP_002273838.1 (VIT_13s0067g01960). We designed primers according to the sequence to clone the gene from the cold-tolerant grapevine cultivar ‘Zuoyouhong’, and the cloned gene was named *VvDREB2A* (GenBank Accession number: MH089405.1). Sequence analyses showed that the *VvDREB2A* cDNA was 1068 bp in full length, and encoded 355 amino acids. The molecular weight of VvDREB2A is approximately 38.74 kDa, and its isoelectric point is 4.93. The 125, 162, 224, and 266 amino acids in ‘Zuoyouhong’ were T, L, H, and S, respectively (GenBank Accession number: AZI71501.1), while the corresponding positions in ‘Pinot Noir’ were M, V, R, and G. The sequence contained an AP2 conserved domain (66–119 aa) and belonged to the AP2 family (Figure 1A). The phylogenetic tree showed that VvDREB2A is closely related to *M. domestica* DREB2A (Figure 1B).

### 2.2. VvDREB2A Acts as a Transcription Factor

**Figure 2:**
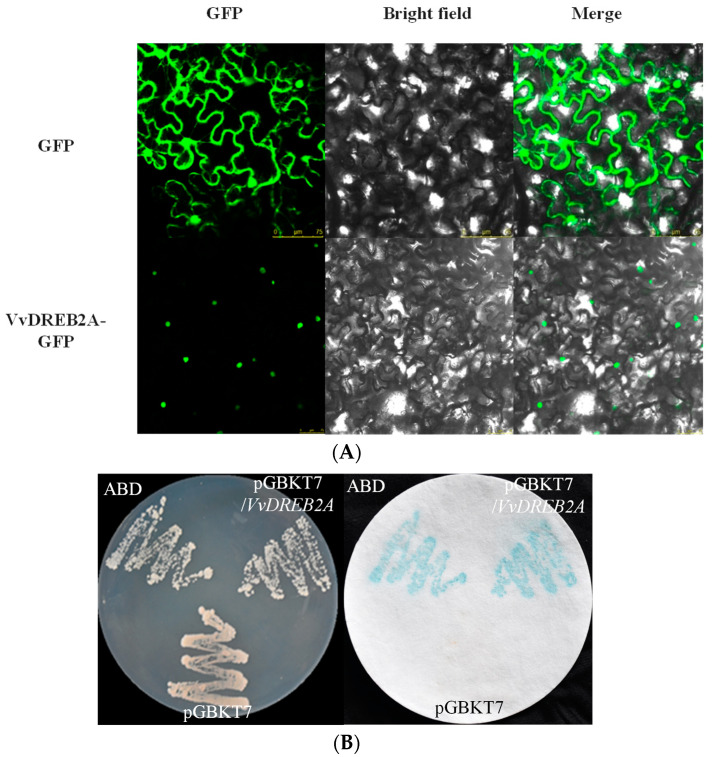
Subcellular localization and transcription activation of VvDREB2A. (**A**) Tobacco leaves were agroinfiltrated with 35S-GFP (control) or 35S-VvDREB2A-GFP (fusion plasmid) and visualized under a confocal microscope after 2 days. From left to right, panels display dark-field images of GFP, bright-field images of cell morphology, and the merged images. (**B**) Yeast cells containing pGBKT7-VvDREB2A, pGBKT7(negative control), and ABD (positive control) were cultured on SD/Trp- medium. X-gal staining was conducted for β-galactosidase activity.

To identify the subcellular localization of VvDREB2A, the pSuper1300-VvDREB2A-GFP was constructed and transiently expressed in tobacco leaves. The results indicated that green fluorescence was only observed in the nucleus of the tobacco leaves (Figure 2A), implying that VvDREB2A was located in the nucleus.

The positive control (ABD) plasmid, the negative control (pGBKT7), and the recombinant plasmid pGBKT7-VvDREB2A were transformed into yeast strain Y187 for the transcriptional activation assay. The positive control and the yeast of the recombinant plasmid pGBKT7-VvDREB2A both turned blue for β-galactosidase activity (Figure 2B), demonstrating that VvDREB2A has transcriptional activity. Therefore, VvDREB2A could probably act as a transcription activator in the nucleus.

### 2.3. VvDREB2A Expression Pattern

**Figure 3:**
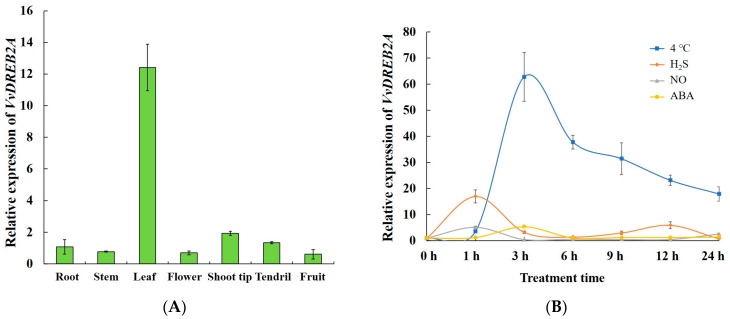
VvDREB2A expression pattern in grapevine. (**A**) *VvDREB2A* expression pattern in different grapevine tissues. (**B**) *VvDREB2A* expression level in response to cold stress and signal molecules. Three repetitious experiments were conducted. Bars are the means ± SD of three repeats.

The expression of *VvDREB2A* in the grapevine cultivar ‘Zuoyouhong’ was detected in different tissues, including the roots, stems, leaves, flowers, the shoot tip, tendrils, and fruit, implying that VvDREB2A was expressed in various tissues and had a relatively high expression level in leaves (Figure 3A). Thus, we speculated that VvDREB2A is mainly involved in leaf-related functions in grapevines.

Further, the tissue culture seedlings of ‘Zuoyouhong’ were subjected to cold stress (4 °C) and cold-related signaling molecules, such as H_2_S, NO, and ABA, were determined. The results showed that 4 °C, H_2_S, NO, and ABA-induced *VvDREB2A* expression. The 4 °C and H_2_S treatments strongly induced *VvDREB2A* expression, which peaked at 3 h and 1 h, respectively (Figure 3B). These data indicate that VvDREB2A is induced by cold stress as well as signaling molecules related to cold stress.

### 2.4. VvDREB2A Enhances Cold Tolerance of Transgenic Arabidopsis

**Figure 4:**
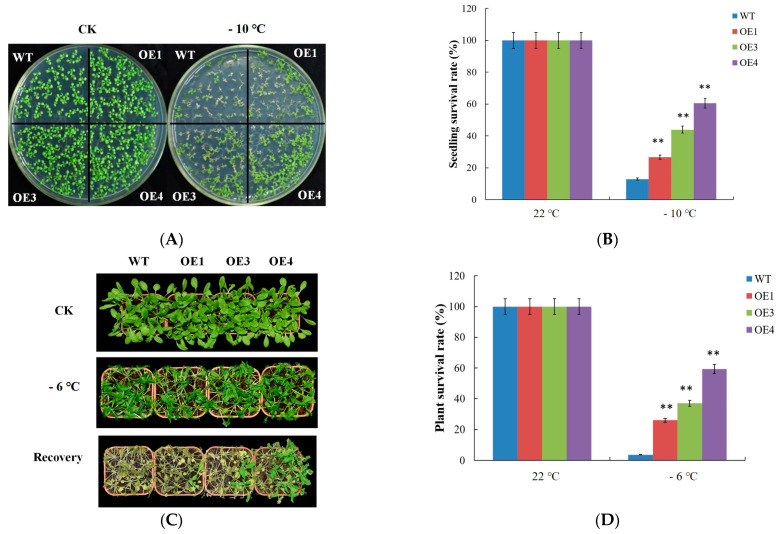
Phenotypes of the *VvDREB2A* transgenic *Arabidopsis* responses to cold stress. (**A**) Phenotypes of 2-week-old *Arabidopsis* seedlings after −10 °C treatment. Each experiment was repeated three times, and there were over 30 seedlings in each line. (**B**) Survival rates of *VvDREB2A*-overexpressing *Arabidopsis* seedlings after the −10 °C treatment. Two asterisks show a significant difference between WT and transgenic lines (** *p* < 0.01). (**C**) Phenotypes of 4-week-old *Arabidopsis* plants under −6 °C treatment. Each experiment was repeated three times, and there were over t 30 seedlings in each line. (**D**) Survival rates of *VvDREB2A* transgenic plants after the −6 °C treatment. Two asterisks show a significant difference between WT and transgenic lines (** *p* < 0.01).

*VvDREB2A* was overexpressed in *Arabidopsis*, and the overexpressing lines were identified by PCR (Supplementary Figure S1A). The relative expression of *VvDREB2A* in transgenic lines was detected by quantitative real-time PCR, and three independent lines (OE1, OE3, and OE4) were screened for subsequent experiments (Supplementary Figure S1B). The 2-week-old WT and *VvDREB2A*-overexpressing *Arabidopsis* seedlings were treated at −10 °C for 1 h, and then cultured at 22 °C for 2–3 days to observe the phenotype and calculate the survival rate. The results showed that the WT and *VvDREB2A*-overexpressing lines had similar growth and development status under normal conditions, while the *VvDREB2A*-overexpressing lines grew better than the WT plants after the −10 °C treatment. We calculated the survival rates of OE1, OE3, and OE4, which were approximately 26%, 43%, and 60%, whereas the survival rate of the WT was approximately 8% (Figure 4A).

The 4-week-old WT and transgenic *Arabidopsis* were subjected to 4 °C for 36 h, followed by −6 °C for 12 h in the dark, then at 4 °C for 12 h, and lastly cultured at 22 °C for 3–4 days to observe the phenotypes. The results showed that the *VvDREB2A*-overexpressing lines grew better than the WT plants after the cold treatment. The survival rates of OE1, OE3, and OE4 were 25%, 37%, and 59% compared to 3% in the WT plants (Figure 4B). These data indicate that VvDREB2A improves cold tolerance in plants.

### 2.5. Overexpression of VvDREB2A Reduces Reactive Oxygen Species Accumulation in Transgenic Arabidopsis under Cold Stress

**Figure 5:**
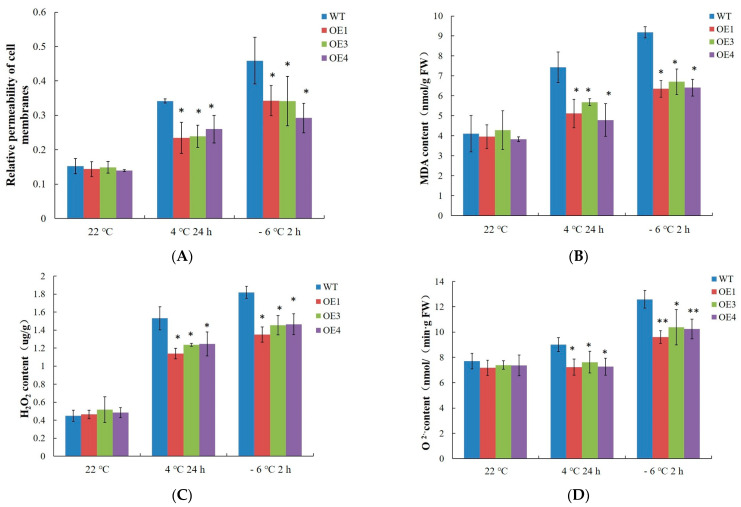
ROS accumulation in *VvDREB2A*-overexpressing *Arabidopsis* under cold stress Relative permeability of the cell membranes (**A**), MDA content (**B**), H_2_O_2_ content (**C**), and O_2_^−^ content (**D**) in the leaves of transgenic *Arabidopsis* after being treated at 4 °C for 24 h or at −6 °C for 2 h. Asterisks show a significant difference between WT and transgenic lines (* *p* < 0.05; ** *p* < 0.01).

Under normal conditions, cell membrane permeability and MDA, H_2_O_2_, and O_2_ contents were not significantly different between the 4-week-old WT and *VvDREB2A*-overexpressing *Arabidopsis*. However, all of these parameters increased in the WT and *VvDREB2A*-overexpressing *Arabidopsis* after the 4 °C and −6 °C treatments, yet the degree of the increase was lower in the *VvDREB2A*-overexpressing lines (Figure 5A–D). These data indicate that *VvDREB2A* improved cold resistance by reducing cell membrane permeability, membrane lipid peroxidation, and the amount of ROS.

**Figure 6:**
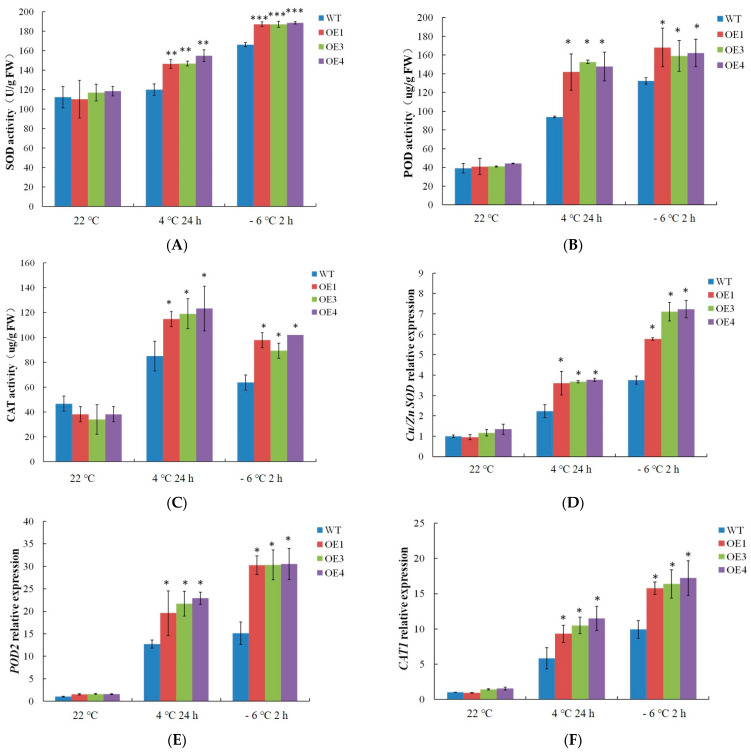
Effects of cold stress on antioxidant enzymes in *VvDREB2A*-overexpressing *Arabidopsis*. The activities of SOD (**A**), POD (**B**), and CAT (**C**), and the expression levels of *Cu/Zn SOD* (**D**), *POD2* (**E**), and *CAT1* (**F**) in transgenic *Arabidopsis* leaves treated at 4 °C for 24 h or at −6 °C for 2 h. Asterisks show significant differences between WT and transgenic lines (* *p* < 0.05; ** *p* < 0.01; *** *p* < 0.001).

As shown in Figure 6, the activity of the antioxidant enzymes was stable under normal conditions, and the genes related to the antioxidant enzymes were accordingly at a low expression level. In contrast, the antioxidant enzyme activity and the related genes (*Cu/Zn SOD*, *POD2*, and *CAT1*) expression levels all increased after the 4 °C and −6 °C treatments, and their levels were higher in the *VvDREB2A*-overexpressing lines compared with WT. These data demonstrated that VvDREB2A enhanced cold resistance by promoting antioxidant enzyme activity.

### 2.6. Overexpression of VvDREB2A Accumulates More Raffinose Family Oligosaccharides under Cold Stress

**Figure 7:**
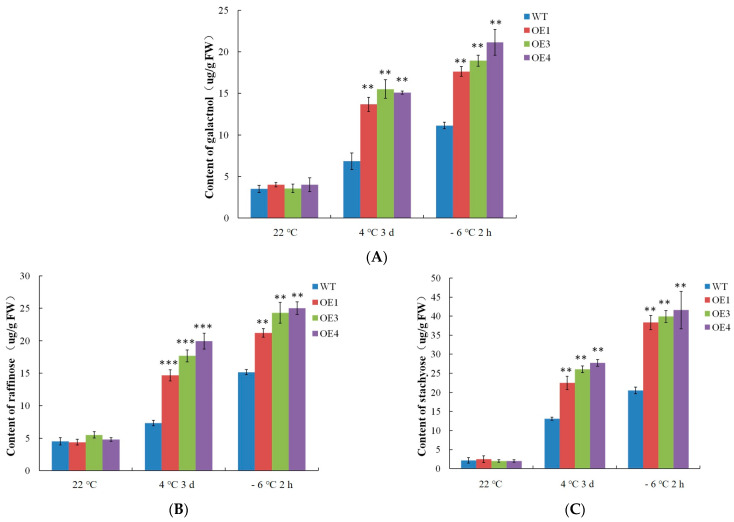
Detection o of raffinose family oligosaccharide contents in *VvDREB2A*-overexpressing lines under cold stress. The amounts of galactinol (**A**), raffinose (**B**), and stachyose (**C**) in transgenic *Arabidopsis* leaves after being treated at 4 °C for 3 days or at −6 °C for 2 h. Asterisks show significant differences between WT and transgenic lines (** *p* < 0.01; *** *p* < 0.001).

The RFOs are the second most abundant class of soluble sugars in higher plants after sucrose. As an important osmotic regulator in plants, RFOs play an important role in responses to abiotic stressors [4]. The synthesis of RFOs begins with raffinose, and the galactose groups on galactinol are transferred sequentially to produce raffinose and stachyose. The RFO contents were detected in this experiment. As shown in Figure 7, the WT and *VvDREB2A*-overexpressing *Arabidopsis* showed little difference in the levels of galactinol, raffinose, and stachyose at 22 °C. Nevertheless, the contents of galactinol, raffinose, and stachyose increased after the 4 °C and −6 °C treatments, and were significantly higher than the WT. As a result, VvDREB2A increases the cold tolerance of plants by increasing RFO contents.

### 2.7. Overexpression of VvDREB2A Promotes Cold-Related Gene Expression

**Figure 8:**
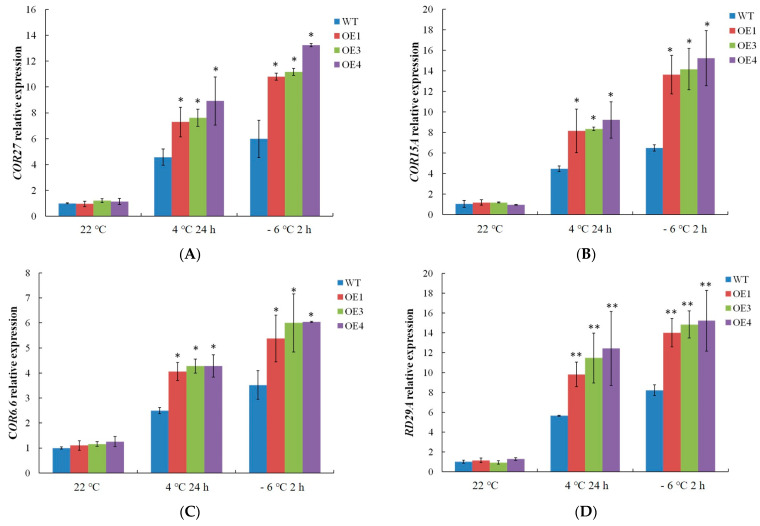
Measurement of cold-related gene expression in *VvDREB2A*-overexpressing lines after cold treatment *COR27* expression (**A**), *COR15A* expression (**B**), *COR6.6* expression (**C**), and *RD29A* expression (**D**) in transgenic *Arabidopsis* leaves after being treated at 4 °C for 24 h or at −6 °C for 2 h. Asterisks show significant differences between WT and transgenic lines (* *p* < 0.05; ** *p* < 0.01).

The expression profiles of the cold-related gene were measured. The expression levels of *COR15A*, *COR27*, *COR6.6*, and *RD29A* in WT and *VvDREB2A*-overexpressing *Arabidopsis* were not significantly different at 22 °C. Meanwhile, the cold-related gene expression levels increased after the 4 °C and −6 °C treatments, and the gene expression levels in the transgenic *Arabidopsis* were markedly higher than those in the WT plants (Figure 8), indicating that *VvDREB2A* enhances cold resistance by increasing cold-related gene expression.

## 3. Discussion

DREB2A is a member of the AP2 transcription factor family. It binds to the CRT/DRE response element of downstream genes to regulate stress-related gene expression and participate in the transduction of abiotic stress signals. The alignment analysis of the *VvDREB2A* gene (MH089405) from the grapevine cultivar “Zuoyouhong” and *VIT_13s0067g01960* from the grapevine cultivar “Pinot Noir” revealed 99% similarity. The amino acid sequence alignment indicated that VvDREB2A belonged to the A2 subfamily, which was consistent with the sequence-based protein classification predicted by Zhao et al. The conservative region of VvDREB2A contained seven key amino acid residues, including one valine (V) residue (68th), two tryptophan (W) residues (76th and 90th), and four arginine (R) residues (66th, 69th, 81st, and 88th) (Figure 1A). These amino acids may directly interact with the DREB protein and the CRT/DRE cis element, which agrees with some previous reports [9,10]. Our subcellular localization and transcriptional activity analyses (Figure 2) proposed that VvDREB2A may function as a transcription factor.

Research on the function of the DREB2 transcription factor family has mainly focused on drought, salinity, and high-temperature resistance. Some transcription factors from this family are widely involved in drought stress, such as AtDREB2A from *Arabidopsis* [8], GmDREB2A from soybean [20], VuDREB2A from cowpea beans [21], and FpDREB2A from *Fraxinus pennsylvanica* [1]. Other members, such as VrDREB21 from green beans [22] and OsDREB2A from *Oryza sativa* [23,24], may be multi-functional and play roles in the salt and drought stress processes. AtDREB2A in *Arabidopsis* binds to the *AtHsfA3* promoter and regulates the response to heat stress [25]. A few DREB2 family members participate in cold stress. The *CpDreb2*-type gene from *Carica papaya* L is expressed under cold stress, and *CpDreb2* ectopic overexpression in tobacco shows an increased amount of proline and greater tolerance under cold stress [16]. *PeDREB2L* from *Populus euphratica Oliva* was transformed into *Arabidopsis* and the transgenic lines showed improved tolerance against cold stress [15]. In this research, *VvDREB2A* was regulated by cold stress and cold-related signaling molecules (Figure 3B). We used *VvDREB2A* ectopically overexpressing *Arabidopsis* as the material and observed that transgenic *Arabidopsis* had a greater tolerance to cold stress and a higher survival rate (Figure 4). These data demonstrated that VvDREB2A enhanced the cold tolerance of plants.

Furthermore, we explored the underlying mechanism by which *VvDREB2A* is involved in cold resistance. According to literature reports, potato (*Solanum tuberosum* L.) *StDREB2*-overexpressing cotton plants decrease ROS content by enhancing the activities of the antioxidant enzymes SOD, CAT, POD, and GST as well as the expression of related genes. As a result, transgenic cotton has improved drought resistance [26]. *PgDREB2A* was cloned from *Pennisetum glaucum* and the *PgDREB2A*-overexpressing *Arabidopsis* showed tolerance to osmotic, salt, cold, and heat stress by alleviating the degree of membrane damage, and increasing proline content [27]. We found that cell membrane permeability, and MDA, H_2_O_2_, and O_2_ contents of the *VvDREB2A*-overexpressing lines decreased significantly (Figure 5), and antioxidant enzyme activities and related gene expression levels increased significantly (Figure 6). These data suggest that *VvDREB2A* alleviated cell damage and improved cold resistance by enhancing antioxidant enzyme activities and reducing ROS content.

Notably, the RFO content increased during cold stress in the *Arabidopsis* lines with heterologous overexpression of *VvDREB2A* (Figure 7). As important osmotic regulators in plants, RFOs respond to abiotic stress [4]. In contrast, RFOs also protect cells from oxidative damage. They could scavenge ROS in the vicinity of organellar membranes so as to maintain cellular ROS homeostasis [28]. Chai et al. measured the metabolic changes in *V. amurensis* and *V. vinifera cv.* grapevine plants after a 4°C treatment and found that galactinol and raffinose contents increased [29]. Sun et al. reported that the grape transcription factor AQUILO increases low-temperature tolerance by increasing raffinose content in transgenic *Arabidopsis* and the grape callus [30]. In our study, the RFO contents increased in the *VvDREB2A*-overexpressing lines under cold stress, demonstrating that VvDREB2A improves cold tolerance by enhancing RFO contents. Further exploration is needed to determine whether VvDREB2A can regulate the genes related to raffinose synthesis and improve the cold tolerance of grapevine.

It is well known that DREB binds to the DRE/CRT motif, which activates the transcriptional expression of downstream genes and participates in the resistance to abiotic stress process [9,31,32]. However, whether VvDREB2A regulates cold-related gene expression is unknown. Therefore, we measured the expression level of the downstream genes. Higher expression of these cold response-related genes was detected compared with wild type, suggesting that VvDREB2A improves cold tolerance through enhancing these marker genes expressions in the transgenic *Arabidopsis*.

## 4. Materials and Methods

### 4.1. Plant Growth and Treatment Conditions

Grapevine tissue culture seedling, *Arabidopsis*, and *Nicotiana tabacum* cultivation: under light conditions of 200 μmol·m^−2^·s^−1^ (12 h/12 h light cycle, 25 ± 1 °C), the grapevine variety ‘Zuoyouhong’ tissue culture seedlings were inoculated on root media (1/2 MS + 0.1 mg·L^−1^ IAA) and continuously cultured for 45–55 d. *Arabidopsis* seeds were sterilized and placed at 4 °C for 2 days to break dormancy, after which they were sown on MS solid medium and incubated in light conditions (16 h/8 h light cycle, 22 ± 2 °C) for 14 days. Then, the seedlings were planted in nutrient-rich soil. *N. tabacum* was grown at 25 °C and with a 12 h/12 h light cycle for the subcellular localization assay.

Grapevine tissue culture seedling treatment: Grapevine tissue culture seedlings growing for 45–55 days were subjected to 4 °C for the cold stress, an H_2_S donor sodium hydrosulfide (NaHS, 0.1 mmol/L), a NO donor sodium nitroprusside (SNP, 0.1 mmol/L), and abscisic acid (ABA, 10 μmol/L) for 0, 1, 3, 6, 9, 12 and 24 h. The seedlings’ leaves were collected to measure the expression level of *VvDREB2A*.

*Arabidopsis* treatment: 2-week-old *Arabidopsis* were treated at −10 °C for 1 h, and then cultured at 22 °C for 2–3 days to observe the phenotypes and collect the survival rate data; 4-week-old *Arabidopsis* were treated at 4 °C for 36 h, followed by the −6 °C dark treatment for 12 h, 4 °C treatment for 12 h, and then cultured at 22 °C for 3–4 days for phenotypic observations and collecting the survival rate data; 4-week-old *Arabidopsis* were treated at 4 °C for 24 h (chilling treatment), or at 4 °C for 36 h followed by −6 °C for 2 h (freezing treatment). Cell membrane permeability; the H_2_O_2_, O_2_^−^, and malondialdehyde (MDA) amounts; antioxidant enzyme activities;and cold response genes expression levels were detected. 4-week-old *Arabidopsis* were treated at 4 °C for 3 days (chilling treatment), or at 4 °C for 36 h followed by −6 °C for 2 h (freezing treatment). The galactinol, raffinose, and stachyose contents were detected.

### 4.2. Cloning and Sequence Analysis of VvDREB2A

The AtDREB2A (AT2G40340) sequence of *Arabidopsis* was subjected to WU-BLAST alignment to search for homologous sequences (XP_002273838.1) from the grapevine cultivar ‘Pinot Noir’. According to the sequences, we designed primers to clone the gene from the cold-tolerant grapevine cultivar ‘Zuoyouhong’. The forward primer was 5′-GCGGATCCATGTCGTCCGGAGTCAT-3′, *Bam*H I site underlined. The reverse primer was 5′-CGAGCTCTTAGAACCCCATATCTGATA-3′, *Sac* I site underlined. The PCR program was 94 °C for 5 min; 30 s at 94 °C, 15 s at 56 °C, and 70 s at 72 °C, 35 cycles; 72 °C for 10 min, and at 4 °C for ∞. The physical and chemical properties of AtDREB2A were analyzed by ProtParam online software. The evolutionary tree was constructed via MEGA 5.0 software with the neighbor-joining method.

### 4.3. Subcellular Localization of VvDREB2A

The full-length cDNA of *VvDREB2A* was amplified with 5′-TCTAGAATGTCGTCCGGAGTCAT-3′ (FP) and 5′-ACTAGAGAACCCCATATCTGATA-3′ (RP), and the amplified sequence was inserted into the pSuper1300-GFP vector driven by a CaMV 35S promoter. The recombinant plasmid pSuper1300-VvDREB2A-GFP was transformed into *Agrobacterium tumefaciens* strain EHA105. Transient expression of the fluorescent protein in tobacco leaf cells was performed as described previously [33]. GFP fluorescence was detected at 488 nm by a laser confocal scanning microscope (SP5, Leica, Wetzlar, Germany).

### 4.4. Transcriptional Activation of VvDREB2A

The primers for transcriptional activation assays were as follows: pGBKT7-VvDREB2A-F, 5′-CCCATGGATGTCGTCCGGAGTCAT-3′, Nco I site underlined, pGBKT7-VvDREB2A-R, 5′-GCGGATCCTTAGAACCCCATATCTGATA-3′, BamH I site underlined. The construct, as well as pGBKT7 (the negative control) and ABD (the positive control), were transformed into yeast strain Y187 on SD/Trp- medium plates. The growth of the transformants on SD/Trp- medium plates was observed after 3 days. A β-galactosidase activity was detected based on the manufacturer’s instructions (Clontech, Palo Alto, CA, USA).

### 4.5. RNA Extraction and qRT-PCR Assay

Total RNA was extracted using the CTAB method according to our previous work [34]. Quantitative real-time PCR was performed at 95 °C for 60 s; 40 cycles of 95 °C for 30 s, 56 °C for 30 s, and 72 °C for 30 s. The genes relative expression was analyzed by the 2^−ΔΔCT^ method. The primers are shown in Table S1 (Supplementary data).

### 4.6. Transformation of Arabidopsis

The *VvDREB2A* fragment was obtained after the pMD18-T-VvDREB2A plasmid was digested with *Bam*H I and *Sac* I. The pSuper1300 vector was digested with the same restriction endonuclease, and the vector was recovered. Then, the *VvDREB2A* fragment and the pSuper1300 vector were ligated to construct the pSuper1300-VvDREB2A. *Agrobacterium tumefaciens* GV3101 containing a recombinant plasmid was transformed into *Arabidopsis* using the floral-dip method. The homozygous T3 progeny were named OE1, OE3, and OE4.

### 4.7. Determination of Physiological Indicator

O_2_ and H_2_O_2_ contents were detected using the methods in our previous work [35]. SOD, POD, and CAT activities were measured using the method of Wang et al. [36]. MDA content and electrolyte leakage were detected according to Fu et al. [37]. Each experiment was repeated three times, and there were at least 30 seedlings in each line.

### 4.8. Determination of Raffinose Family Oligosaccharide Contents

Galactinol, stachyose, and raffinose were extracted from *Arabidopsis* as described by Sui et al. [38]. Plant leaves were pulverized in liquid nitrogen, then 1 mL of 80% ethanol (*v*/*v*) was added and placed at 85 °C for 30 min. After centrifugation at 14,000 rpm for 20 min, the supernatant was collected, then extracted twice with 80% ethanol, dried at 42 °C, and the solid precipitate was dissolved in 1 mL of ddH_2_O. The RFOs were quantified by high-performance liquid chromatography (HPLC), according to Sui et al. [38]. Each experiment was repeated three times, and there were at least 30 seedlings in each line.

### 4.9. Statistical Analysis

All physiological data were calculated by the *t*-test via SPSS software (SPSS Inc., Chicago, IL, USA). A *p*-value < 0.05 was regarded as significant differences.

## 5. Conclusions

In summary, we cloned *VvDREB2A* from grapevine cultivar ‘Zuoyouhong,’ and its GenBank accession number is MH089405.1. The amino acids sequence contained an AP2 conserved domain and belonged to the AP2 family. Subcellular localization and transcriptional activation assays indicated that VvDREB2A acted as a transcription factor. *VvDREB2A* was significantly induced by cold and the stress-signaling molecules. Moreover, *VvDREB2A* plays a positive regulatory function under cold stress by alleviating the levels of ROS, increasing RFO content, and enhancing the expression of cold stress response genes.

## Data Availability

Data are contained within the article or Supplementary Materials.

## Supplementary Materials

The following supporting information can be downloaded at: https://www.mdpi.com/article/10.3390/ijms24119381/s1.
