# Supplementary material for: Transcription Factor VvDREB2A from Vitis vinifera Improves Cold Tolerance
Source: Int J Mol Sci. 2023 May 27;24(11):9381. doi: 10.3390/ijms24119381 (PMC10253714; doi:10.3390/ijms24119381)
Supplement: Supplementary file 1 [file ijms-24-09381-s001.zip › Supplementary Materials.pdf]

Supplementary Materials:

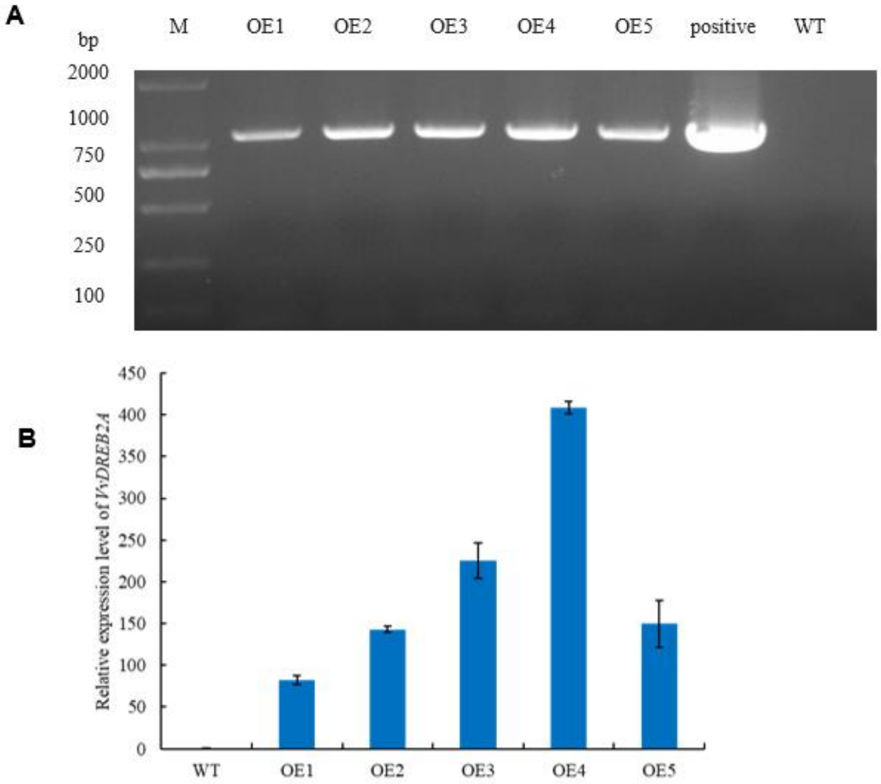

Supplementary Figure 1. Identification of the *VvDREB2A* transgenic *Arabidopsis;* (**A**). PCR identification of *VvDREB2A*-overexpressing *Arabidopsis* (**B**). qRT-PCR identification of *VvDREB2A*-overexpressing *Arabidopsis.*

Supplementary Table 1. Real-time PCR primers.

Gene Name	Primer Sequence(5′ to 3′)
VvDREB2A	FP: ATGGTTCTTCTGCCCGTCTT<br>RP: ACCGCTTATTCCTGATTCACC
VvACTIN	FP: ATAGAAGCAGCAAGGGA<br>RP: TGAGGCTCTTACTAATG
AtCu/ZnSOD	FP: CTGCATCTCTACTGGACCTC<br>RP: CCACATCCAACTCTCGAGC
AtPOD2	FP: GTTTACCCGACCCTACACTC<br>RP: ATCCTATTCATTGCCTCCAC
AtCAT1	FP: GTCCTGGGATTCAGACAGGC<br>RP: GGCCTCACGTTAAGACGAGT
AtRD29A	FP: GGTTGGGAGGATTAAAGGATG<br>RP: AACAGTGGAGCCAAGTGATTG
AtCOR15A	FP: GCTTCAGATTTCGTGACGGATAAAAC<br>RP: GCAAAACATTAAAGAATGTGACGGTG
AtCOR6.6	FP: AAAGCAGAGTGGTGTTGGTACCGT<br>RP: TCATCGAGGATGTTGCCGTCACTT
AtCOR27	FP: CAGTGTCGGAGAGTGTGGTG<br>RP: ACAGCTGGTGAATCCTCTGC
AtACTIN	FP: GGTAACATTGTGCTCAGTGGTGG<br>RP: CACGACCTTAATCTTCATGCTGC
