# Supplementary figures and images for: Transcription Factor VvDREB2A from Vitis vinifera Improves Cold Tolerance
Source: Int J Mol Sci. 2023 May 27;24(11):9381. doi: 10.3390/ijms24119381 (PMC10253714; doi:10.3390/ijms24119381)

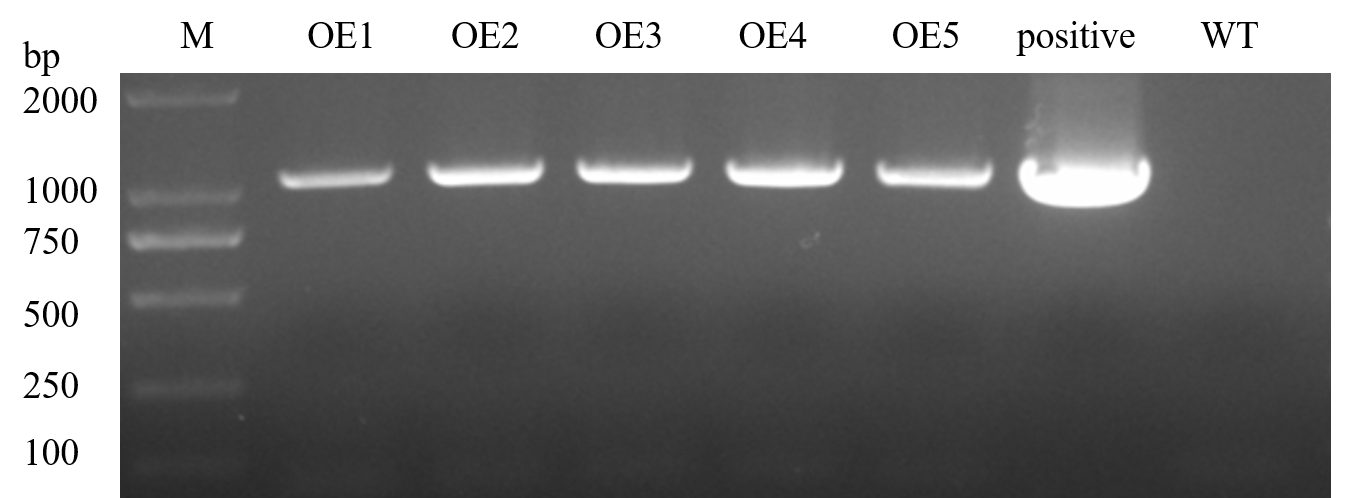

Supplement: Supplementary file 1 [file ijms-24-09381-s001.zip › Fig S1A.tif]

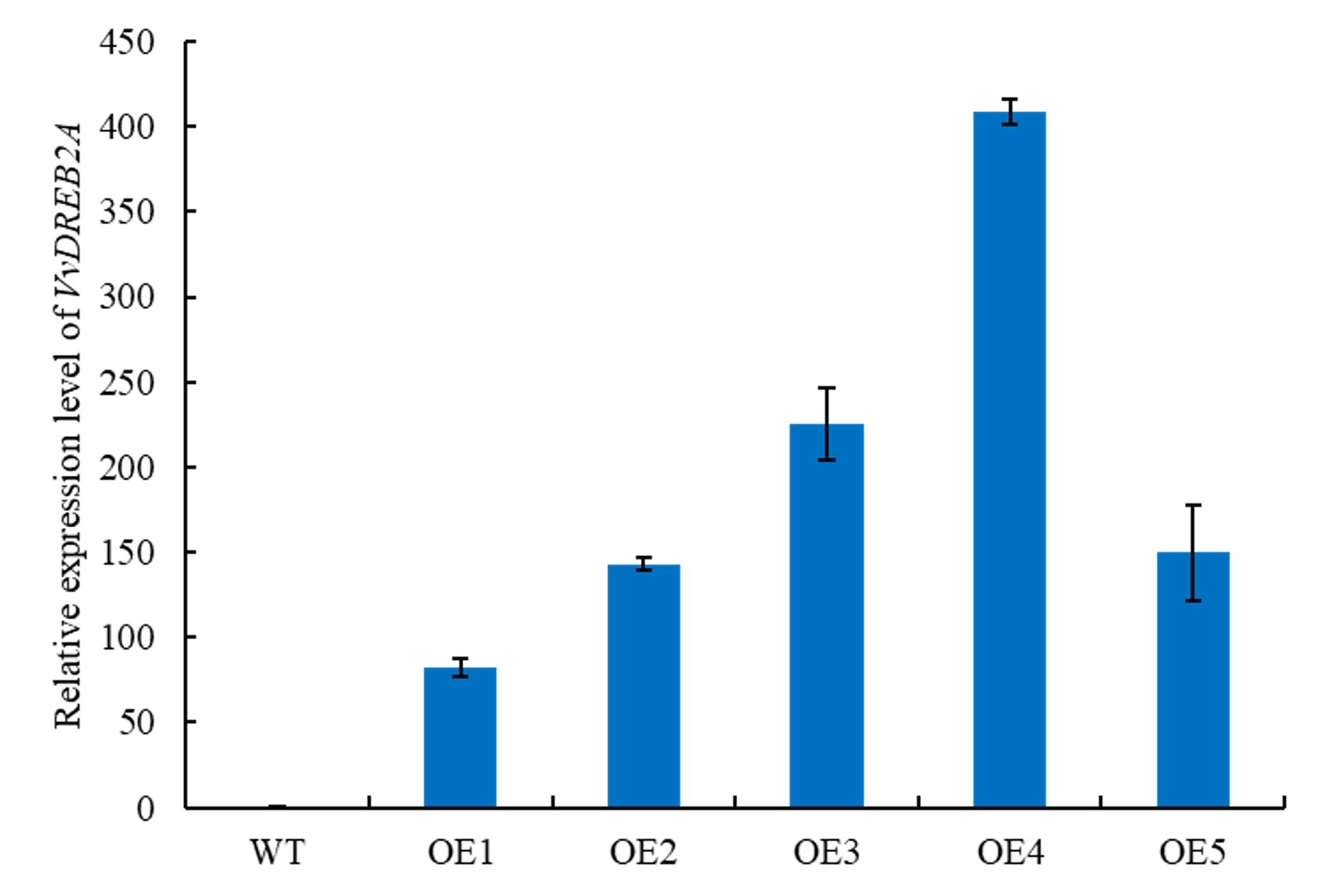

Supplement: Supplementary file 1 [file ijms-24-09381-s001.zip › Fig S1B.tif]
